# Identification of critical residues in Gap3 of *Streptococcus parasanguinis *involved in Fap1 glycosylation, fimbrial formation and *in vitro *adhesion

**DOI:** 10.1186/1471-2180-8-52

**Published:** 2008-03-27

**Authors:** Zhixiang Peng, Paula Fives-Taylor, Teresa Ruiz, Meixian Zhou, Baiming Sun, Qiang Chen, Hui Wu

**Affiliations:** 1 Departments of Pediatric Dentistry and Microbiology, University of Alabama at Birmingham Schools of Dentistry and Medicine, Birmingham, AL 35294, USA; 2 Department of Microbiology and Molecular Genetics, University of Vermont, Burlington, VT 05405, USA; 3 Department of Endodontics, Guanghua College of Stomatology, Sun Yat-Sen University, Guangzhou, 510055, China; 4 Molecular Physiology and Biophysics, University of Vermont, Burlington, VT 05405, USA

## Abstract

**Background:**

*Streptococcus parasanguinis *is a primary colonizer of human tooth surfaces and plays an important role in dental plaque formation. Bacterial adhesion and biofilm formation are mediated by long peritrichous fimbriae that are composed of a 200 kDa serine rich glycoprotein named Fap1 (fimbriae-associated protein). Glycosylation and biogenesis of Fap1 are modulated by a gene cluster downstream of the *fap1 *locus. A gene encoding a glycosylation-associated protein, Gap3, was found to be important for Fap1 glycosylation, long fimbrial formation and Fap1-mediated biofilm formation.

**Results:**

Deletion and site-directed mutagenesis were employed to dissect the regions within Gap3 that were important for its function in Fap1 glycosylation and biogenesis. A deletion of 6 consecutive amino acids, PDLPIL, eliminated the production of the mature 200 kDa Fap1 protein and gave rise instead to a 470 kDa Fap1 intermediate that was only partially glycosylated. Site-directed mutagenesis of the 6 amino acids revealed that only three of these amino acids were required. Mutants in these amino acids (L64R, P65R and L67T) produced the premature 470 kDa Fap1 intermediate. Mutants in the remaining amino acids produced the mature form of Fap1. Cell surface expression of the Fap1 precursor among L64R, P65R and L67T mutants was reduced to levels consistent with that of a *gap3 *insertional mutant. Electron micrographs showed that these 3 mutants lost their long peritrichous fimbriae. Furthermore, their *in vitro *adhesion ability to saliva-coated hydroxylapatite (SHA) was inhibited.

**Conclusion:**

Our data suggest that 3 highly conserved, hydrophobic residues L64, P65 and L67 in Gap3 are essential for Gap3 function and are important for complete glycosylation of Fap1, fimbrial formation and bacterial adhesion.

## Background

*Streptococcus parasanguinis *is an early colonizer of human tooth surface [1,2] and functions for the subsequent colonization of other oral bacteria. The accumulation of oral microorganisms on the tooth surfaces leads to the development of a structured microbial community known as dental plaque, one of the most common human biofilms [3-5].

The adhesion of *S. parasanguinis *FW213 to an *in vitro *tooth model is mediated by its long, peritrichous fimbriae [6,7]. A fimbriae-associated adhesin, Fap1, is the major subunit of the long fimbriae [8]. Fap1, a serine-rich glycoprotein, is required for biofilm formation and bacterial adhesion to saliva-coated hydroxylapatite (SHA, an *in vitro *tooth model [8,9]). A *fap1 *genomic island has been identified in the *S. parasanguinis *FW213 genome. This island consists of seven genes, *secY2*, *gap1 *to *gap3*, *secA2*, *gtf1*, and *gtf2 *and has been shown to play an important role in Fap1 glycosylation and biogenesis [10,11]. Moreover, gene clusters similar to the *fap1 *locus have been identified in other Gram-positive bacteria such as *Streptococcus gordonii, Streptococcus sanguinis, Streptococcus pneumoniae*, *Staphylococcus aureus*, *Staphylococcus epidermidis*, and *Streptococcus agalactiae *[12-17]. The findings that the *fap1*-like loci are widespread among Gram-positive bacteria suggest that there is a common mechanism in the biogenesis of serine-rich glycoproteins. Therefore, the study of the *fap1 *locus will help us to understand the molecular mechanism underlying the biogenesis of Fap1-like molecules.

In this study, we characterized a Fap1 glycosylation-associated gene *gap3 *and identified three amino acid residues in Gap3 that are essential for mature Fap1 glycosylation and biogenesis. We also determined the effects of the *gap3 *mutagenesis on *S. parasanguinis *fimbriae formation and bacterial adhesion.

## Results

### Identification of a small conserved region that is required for Gap3 function

**Figure 1:**
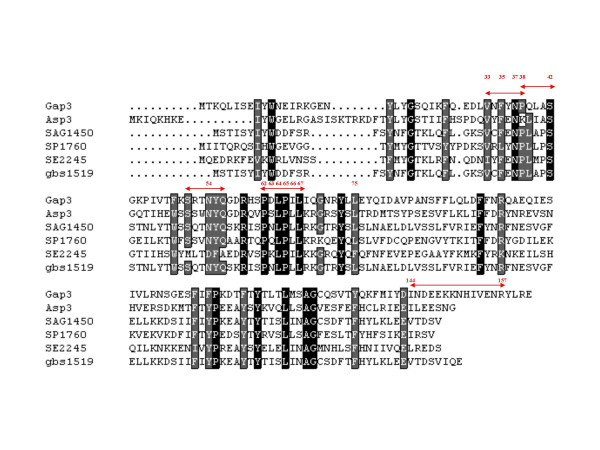
Alignment of Gap3 and its homologues. The black box represents identical or highly conserved residues. The gray box represents similar or less conserved residues. The red arrowed lines indicate the putatively important regions. The red numbers represent the residues chosen for site-directed mutagenesis. Gap3: glycosylation associated protein-3 of *S. parasanguinis*. Asp3: accessory secretory protein-3 of *S. gordonii*. SAG1450: hypothetical protein of *S. agalactiae*. SP1760: hypothetical protein of *S. pneumoniae*. SE2245: hypothetical protein of *Staphylococcus epidermidis*. gbs1519: hypothetical protein gbs1519 of *S. agalactiae*.

**Figure 2:**
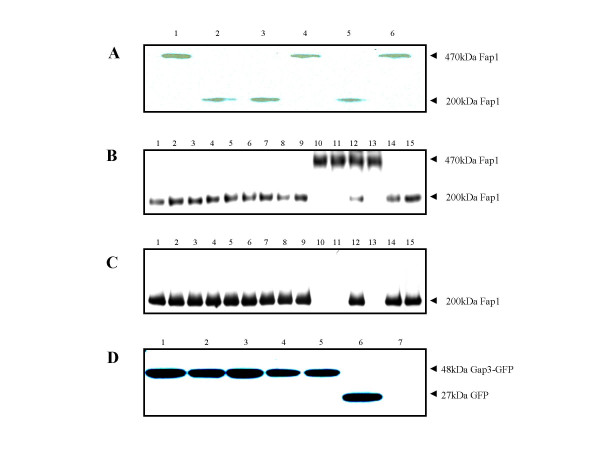
**Western blot analysis of expression of Fap1 and Gap3 variants**. Gap3 deletion mutants, Δ62–67 mutant (Lane 1), Δ144–157 mutant (Lane 2), wild type strain (Lane 3), *gap3 *allelic replacement mutant VT1619 (Lane 4), *gap3 *complemented strain VT1732 (Lane 5) and a control strain (VT1619 transformed with empty vector pVT1666) (Lane 6) were subjected to Western blot analysis with the use of MAb D10 (A). Expression of Fap1 by Gap3 site-directed mutants, V33R, F35H, N37I, P38R, S42L, N54I, R59L, P62G, D63V, L64R, P65R, I66N, L67T and L75S (Lanes 1–14) and wild type strain (Lane 15) probed with MAb D10 (B) and MAb F51 (C). Expression of Gap3-GFP by *gap3 *site-directed mutants, Gap3 L64R (Lane 1), P65R (Lane 2), I66N (Lane3), L67T (Lane4), Gap3 complemented strain VT1732 (Lane 5), control (VT1619 with empty vector pVT1666) (Lane 6) and *gap3 *mutant VT1619 (Lane 7) probed with anti-GFP MAb (C). Gap3-GFP fusion proteins migrate at 48 kDa; GFP protein migrates at 27 kDa.

Gap3 and its homologues in Gram-positive bacteria, Asp3 of *S. gordonii*, SAG1450 and gbs1519 of *S. agalactiae*, SP1760 of *S. pneumoniae *and SE2245 of *Staphylococcus epidermidis*, were identified from the corresponding genomes and aligned to determine the conserved regions (Fig. 1); a search of protein databases was carried out to predict functional significance (whether they are carbohydrate-biosynthesis related) of the conserved sequences using online bioinformatics programs. A series of in-frame deletion mutants and site-directed mutants of *gap3 *were constructed to investigate if these conserved or putatively functional regions/residues were required for Gap3 function and involved in Fap1 biogenesis. One region contains the peptide sequence of PDLPIL (residues 62 to 67), which was also found in a signal receiver domain of a bacterial polysaccharide biosynthesis regulator protein, GelA, of *Sphingomonas elodea *[18]; another region has a peptide sequence of INDEEKKNHIVENR (from residue 144 to 157 of Gap3), which has a putative coiled-coil domain as predicated by an online program [19]. We hypothesized the two regions are functionally relevant to Fap1 glycosylation. Interestingly, deletion of the highly conserved region, Δ62–67, inhibited the production of the 200 kDa mature Fap1 (Fig. 2A, Lane 1), whereas deletion of the predicted coiled-coil domain from amino acid residues 144 to 157 did not affect mature Fap1 production (Fig. 2A, Lane 2), suggesting the amino acid residues from 62 to 67, PDLPIL were important for Gap3 function in Fap1 glycosylation.

### Identification of Gap3 residues that affect mature Fap1 production

To define the critical residues responsible for Gap3 function, we used a site-directed mutagenesis scheme to change all amino acids in this 62PDLPIL67 sequence. We also mutated some other conserved amino acids (V33, F35, N37, P38, S42 and N54), the amino acid residues derived from three conserved regions, 33VNFYNP38, 37 NPQALS42 and 51SRTNYQ56. These conserved regions are also present in gene products that are relevant to protein glycosylation. 33VNFYNP38 was found in Beta-glucosidase BgIC of *Thermomonospoa fusca *(EMBL, AF086819); 37NPQLAS42 was found in both glycogen phosphorylase of *Fusobacterium nucleatum *(EMBL, AABF01000011) and CDP-diacylglycerol – glycerol-3-phosphate 3-phosphatidyl-transferase of *Synechocystis sp*.(EMBL, BA000022); whereas 51SRTNYQ56 is observed in a Glucan 1,4-alpha-maltohexaosidase of *Exiguobacterium sibiricum *(EMBL, AADW02000010). Therefore, we generated the conserved residues directed mutants and determined the functional contribution of these conserved residues to Gap3 activity and Fap1 glycosylation. Three Fap1 specific MAbs, including two glycan-specific antibodies F51 and D10 and one peptide-specific antibody E42 [20] were used to examine the effect of the *gap3 *mutation on Fap1 production. Two forms of Fap1 were identified, the mature 200 kDa Fap1, recognized by all three MAbs and a 470 kDa Fap1 recognized only by E42 and D10 [10,21]. The 470 kDa Fap1 precursor, found in some *gap3 *mutants, has been identified as a partially glycosylated form of Fap1 [21-23]. Most *gap3 *site-directed mutants, V33R, F35H, N37I, P38R, S42L, N54I, P62G and D63V retained the ability to produce the 200 kDa mature Fap1 (Fig. 2B and 2C, Lanes 1–8), suggesting that these amino acids in Gap3 are not involved in Fap1 glycosylation. In contrast, 3 site-directed mutants in the PDLPIL region, the L64R, P65R and L67T mutants failed to produce the 200 kDa mature Fap1. Instead they generated the 470 kDa partially glycosylated Fap1 precursor (Fig. 2B and 2C, Lanes 9, 10 and 12). Since both leucine residues 64 and 67 are important for Gap3 function, we also mutated an adjacent leucine residue L75, the L75R mutant did not alter Fap1 glycosylation (Fig. 2B and 2C, Lanes 14), suggesting the importance of L64 and L67 residues is position-specific. Another highly conserved residue near 62PDLPIL67, R59, was also mutated with leucine and was proved to have no effect on Fap1 glycosylation (Fig. 2B and 2C, Lanes 7). Interestingly, mutation of a less conserved residue, I66N, resulted in a production of both the 200 kDa mature Fap1 and the 470 kDa partially glycosylated Fap1 (Fig. 2B and 2C, Lanes 11). To confirm the mutagenesis did not alter the Gap3 stability and expression, the Gap3 site-directed variants were tagged with GFP (Green Fluorescent Protein) and the expression of Gap3-GFP variants was determined by Western blot analysis (Fig. 2D, Lane 1–4). All the mutant variants were expressed at the same level, suggesting that the mutagenesis did not change the stability of Gap3. Therefore we conclude that the three highly conserved amino acid residues are essential for Gap3 function and have an obvious impact on glycosylation of Fap1.

### Cell surface expression of Fap1

**Figure 3:**
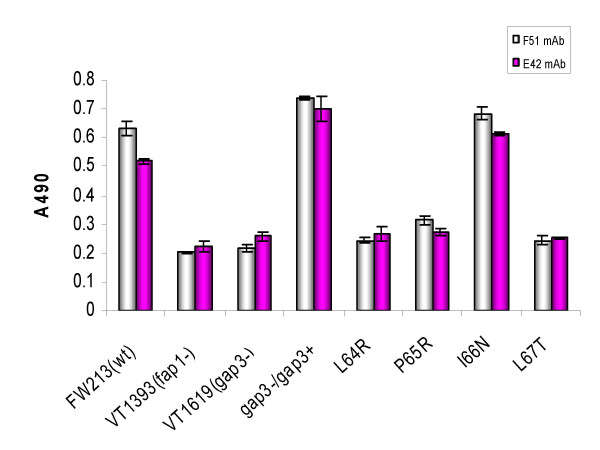
Cell surface expression of Fap1 determined by BactELISA. MAbs F51 and E42 were used in BactELISA analysis to determine Fap1 expression in wild type FW213, *fap1 *mutant VT1393, *gap3 *mutant VT1619, *gap3 *complemented strain *gap3-/gap3+ *and Gap3 site-directed mutants, L64R, P65R, I66N and L67T. Data were obtained from three experiments in triplicates and are presented as means ± standard deviation.

A BactELISA method was used to investigate Fap1 expression on the cell surface. The wild type strain FW213 and the *gap3 *complemented strain VT1732 reacted strongly with Fap1-specific antibodies, MAbs F51 and E42 (Fig. 3). The Gap3 I66N mutant reacted with both F51 and E42, suggesting that I66N mutation did not significantly affect the cell surface expression of mature Fap1. The L64R, P65R and L67T mutants all had low levels of reactivity with MAb E42 in comparison with the *fap1 *null mutant and the *gap3 *insertional mutant. These results suggested that only a minimal amount of partially glycosylated Fap1 precursor was present on the cell surface of the three mutants.

### The surface morphological changes of the *gap3 *mutants

**Figure 4:**
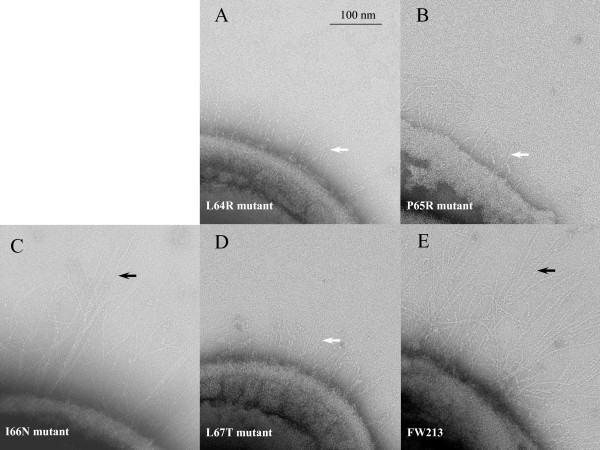
Electron micrographs of *S. parasanguinis *bacteria. *S. parasanguinis *bacteria, L64R (A), P65R (B), I66N (C), L67T (D) mutant variants and wild type FW213 (E) were placed on grids, and negatively stained with 2% phosphotungstic acid pH7.0 and visualized by electron microscopy. White arrows point to the short fimbriae. Black arrows point to the long fimbriae. Scale bar = 100 nm.

Transmission Electron Microscopy (TEM) was used to examine the effects of the site-directed mutagenesis of Gap3 on fimbrial formation of *S. parasanguinis*. Among the 4 site-directed mutants, three mutants L64R, P65R and L67T lost the long peritrichous fimbriae (Fig. 4A, B and 4D), which are characteristic of the wild type strain FW213 [7,24-26], whereas the fourth mutant I66N retained its long fimbriae (Fig. 4C). These data demonstrated the partially glycosylated form of Fap1 in these mutants could not be assembled into the long fimbriae.

### The *in vitro *adhesion assay

**Figure 5:**
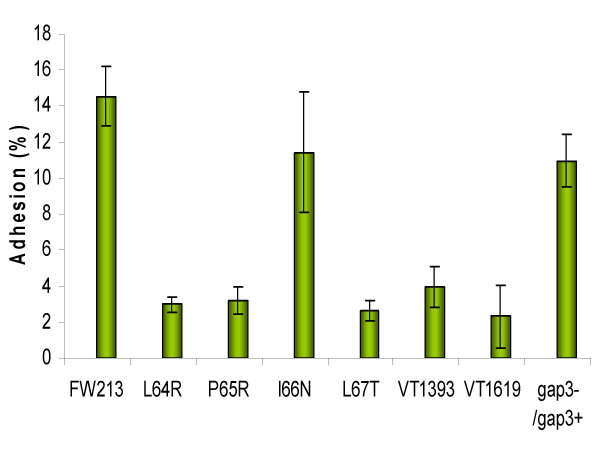
Adhesion of *S. parasanguinis *to saliva-coated hydroxylapatite. Wild type of *S. parasanguinis *FW213, Gap3 site-directed mutants (L64R, P65R, I66N and L67T), *fap1 *mutant VT1393, *gap3 *mutant VT1619 and *gap3 *complemented strain *gap3-/gap3+ *were labeled with [^3^H] thymidine respectively. Labeled cells were incubated with SHA. The amounts of radioactivity associated with beads and supernatants were determined in a Wallac 1400 liquid scintillation counter and calculated to determine adhesion percentage. The data were obtained from three independent experiments in three replicates and are presented as means ± standard deviation.

Fap1 is important for *S. parasanguinis *adhesion [8]. The above data showed that Gap3 site-directed mutation affected Fap1 glycosylation and biogenesis. An adhesion experiment was performed to determine if the partially glycosylated form of Fap1 could still function in adhesion of the Gap3 mutants (Fig. 5). Like *fap1 *and *gap3 *null mutants, the L64R, P65R and L67T mutants failed to adhere to SHA, suggesting the partially glycosylated form was unable to function in bacterial adhesion. I66N had a modest reduction in bacterial adhesion, indicating the I66 mutation only had a minor effect on adhesion.

## Discussion and Conclusion

Fap1, a 200 kDa glycoprotein, is the major subunit of the long fimbriae of *Streptococcus parasanguinis *[8]. A genomic island in the chromosome of *S. parasanguinis *FW213 is involved in Fap1 biosynthesis. This genomic island contains a seven-gene cluster located immediately downstream of *fap1*. This cluster includes *secY2, gap1, gap2, gap3 *(formerly *orf1 *to *orf3*), *secA2*, *gtf1 *and *gtf2 *that are differentially involved in Fap1 glycosylation and secretion [10,11]. Our previous studies showed that the gene product Gap3 is involved in Fap1 glycosylation [23]. In this study, we determined the critical amino acid residues that are important for its function. We first aligned Gap3 and its homologues to find the conserved regions and then took bioinformatics approaches to further analyze functional domains. A conserved peptide sequence, 62PDLPIL67 was found to be critical for Gap3 function and Fap1 glycosylation. This sequence domain was also found in a signal receiver domain of a bacterial polysaccharide biosynthesis regulator protein, GelA, of *Sphingomonas elodea *(NCBI, AAP46184). This may explain why this region is important for Fap1 glycosylation. Interestingly, the other predicted important peptide sequence from residue 144 to 157 of Gap3 is not required for Fap1 glycosylation despite the fact it has a putative coiled-coil structure (a potential protein-protein interaction domain). Therefore, we conclude the 62PDLPIL67 sequence is critical for Gap3 function.

Site-directed mutagenesis was used to replace every amino acid in the sequence 62PDLPIL67 by a disfavored amino acid [27] to determine which amino acid(s) in this region are important for Fap1 glycosylation. L64R, P65R and L67T mutants failed to produce fully-glycosylated mature Fap1, indicating the amino acids L64, P65 and L67 are critical for the complete glycosylation of Fap1. The replacement of the hydrophobic residues L64, P65 and L67 by charged and hydrophilic residues (R and T) did not change overall Gap3 structure as all these mutant variants expressed Gap3 equally (Fig. 2D). These findings highlight the importance of three residues for Gap3 function.

Site-directed mutagenesis of 7 other conserved amino acids V33, F35, N37, P38, S42, N54 and R59 did not alter Fap1 glycosylation despite these amino acids (with the only exception of R59) residing in putative conserved functional domains. Mutagenesis of an adjacent leucine residue L75 did not change Fap1 glycosylation either. These results support the notion that these three important residues identified in 62PDLPIL67 region are site-specific in Gap3 function.

The mechanism for how this region is involved in Gap3 function currently is unknown. Many hydrophobic residues are functionally important in the protein-protein interaction processes [27]. The PDLPIL region has two proline and two leucine residues, both are hydrophobic amino acids and have been shown to play important roles in molecular- or substrate-recognition through hydrophobic interactions [27-29]. In this study, we replaced the conserved, hydrophobic amino acids with hydrophilic or charged, polar amino acids whose properties are totally different (L64R, P65R and L67T). The site-directed mutagenesis resulted in the attenuation of Gap3 activity in Fap1 complete glycosylation. Interestingly, the mutagenesis of a less conserved amino acid, I66 in the same region, had only a minor effect on mature Fap1 production, suggesting that I66 is less critical in Gap3 function and may not be crucial in mediating protein-protein interactions that are important for Gap3 mediated Fap1 glycosylation.

Fap1 is a structural subunit of bacterial long fimbriae and essential for fimbrial assembly and bacterial adhesion [8]. In this study, we demonstrated that the partially glycosylated Fap1 precursor produced by the *gap3 *site-directed mutants, L64R, P65R and L67T could not be assembled into the long fimbriae, suggesting that glycosylation plays an important role in Fap1 mediated fimbrial formation. All mutants that failed to produce mature Fap1 could not adhere well to SHA *in vitro*, further demonstrating the importance of mature Fap1 in bacterial adhesion. Interestingly the I66N mutant that produces both mature and premature Fap1 still displays fimbriae on the cell surface and is able to adhere to SHA, supporting the concept that mature Fap1 is required for fimbrial biogenesis and bacterial adhesion. Understanding the underlying mechanism will help to define Fap1 biogenesis and fimbrial assembly pathways.

Our finding of 3 critical residues in Gap3 function does not preclude the possible roles of other amino acids that were not selected for this study. Gap3 protein is predicted to be involved in a glycosylation-related complex with other Gap proteins (Gap1 and Gap2). Our future investigations will be to determine if there are more active sites in Gap3 associated with protein-protein interaction during Fap1 biogenesis process.

In conclusion, we have identified that 3 highly conserved residues, L64, P65 and L67 of Gap3 that are critical for complete Fap1 glycosylation, long fimbrial formation and bacterial adhesion. Because the glycosylation-association protein is conserved in many other streptococci and staphylococci, and the three residues we identified are also highly conserved, this work may shed a light on further understanding the mechanism of biogenesis of Fap1-like serine-rich glycoproteins.

## Methods

### Bacterial strains, media, growth conditions and antibodies

All bacterial strains, plasmids and antibodies used in this study are listed in Table 1. Frozen *Streptococcus parasanguinis *and its derivatives preserved with 5% dimethyl sulphoxide were streaked onto Todd-Hewitt (TH) agar (Difco Laboratories, Detroit, MI) plates, and incubated for 15–18 hours in the presence of 10% CO_2 _at 37°C. Liquid cultures were prepared by inoculating single colonies from TH agar plates into TH broth and grown statically in 10% CO_2 _at 37°C. Cell concentrations were determined by a growth curve with absorbance at 470 nm. The *E. coli *was cultured in Luria-Bertani (LB) medium (Difco Laboratories, Detroit, MI). Antibiotics were used at the following concentrations: 10 μg/ml erythromycin and 125 μg/ml kanamycin in TH broth or agar plates for *S. parasanguinis*; 500 μg/ml erythromycin and 50 μg/ml kanamycin in LB broth or agar plates for *E. coli*.

**Table 1:** Bacterial strains, plasmids and antibodies used in this study

Strain, plasmid or antibody	Relevant characteristics	Reference or source
Strains		
*S. parasanguinis*		
FW213	Wild type	[7]
VT1393	*fap1::aphA-3*, null mutant of *fap1*, Kan^R^	[8]
VT1619	*gap3 *replacement mutant, Kan^R^	[23]
VT1731	pVT1666 into VT1619, vector control strain, Erm^R ^& Kan^R^	This study
VT1732	pVT1732 into VT1619, *gap3 *complemented strain, Erm^R ^&Kan^R^	This study
VT1735	Gap3 aa 62–67 encoding region deletion in pVT1732, Erm^R ^&Kan^R^	This study
VT1737	Gap3 aa 144–157 encoding region deletion in pVT1732, Erm^R ^&Kan^R^	This study
VT1738	Gap3 V33R mutation of pVT1732 in VT1619, Erm^R ^&Kan^R^	This study
VT1739	Gap3 F35F mutation of pVT1732 in VT1619, Erm^R ^&Kan^R^	This study
VT1740	Gap3 N37I mutation of pVT1732 in VT1619, Erm^R ^&Kan^R^	This study
VT1741	Gap3 P38R mutation of pVT1732 in VT1619, Erm^R ^&Kan^R^	This study
VT1742	Gap3 S42L mutation of pVT1732 in VT1619, Erm^R ^&Kan^R^	This study
VT1743	Gap3 N54I mutation of pVT1732 in VT1619, Erm^R ^&Kan^R^	This study
VT1744	Gap3 P62G mutation of pVT1732 in VT1619, Erm^R ^&Kan^R^	This study
VT1745	Gap3 D63V mutation of pVT1732 in VT1619, Erm^R ^&Kan^R^	This study
VT1746	Gap3 L64R mutation of pVT1732 in VT1619, Erm^R ^&Kan^R^	This study
VT1747	Gap3 P65R mutation of pVT1732 in VT1619, Erm^R ^&Kan^R^	This study
VT1748	Gap3 I66N mutation of pVT1732 in VT1619, Erm^R ^&Kan^R^	This study
VT1749	Gap3 L67T mutation of pVT1732 in VT1619, Erm^R ^&Kan^R^	This study
VT1750	Gap3 L75S mutation of pVT1732 in VT1619, Erm^R ^&Kan^R^	This study
VT1751	Gap3 R59L mutation of pVT1732 in VT1619, Erm^R ^&Kan^R^	This study
*E. coli*		
DH10B	Host strain for cloning	Invitrogen
XL-10-Gold	Host strain for site-directed mutagenesis	Stratagene
Plasmids		
pVT1571	An *E. coli and S. parasanguinis *shuttle vector, parent of pVPT1686	[11]
pVT1666	PVT1571 vector contained *gfp *gene	This study
pVT1732	pVT1666 contained *gap3 *gene	This study
Antibodies		
D10	Partilly glycosylated Fap1 glycan epitope-specific MAb	[20]
E42	Fap1 peptide epitope-specific MAb	[20]
F51	Mature Fap1 glycan epitope-specific MAb	[20]
Anti-GFP	MAb against GFP tag	Roche Company

Several monoclonal antibodies were used to detect Fap1 and its intermediate form [8,25]. Among them, MAb E42 is specific to the peptide epitope in the polypeptide backbone region of Fap1, whereas MAbs F51 and D10 are specific to different glycan epitopes in the carbohydrate-modified region of Fap1 [20]. Research with Fap1 glycosylation-defective mutants reveal the glycan-specific MAb F51 only recognizes the 200 kDa mature Fap1, whereas peptide-specific MAb E42 and another glycan-specific MAb D10 recognize both the 200 kDa mature Fap1 and the 470 kDa partially glycosylated Fap1 precursor [10,20,21].

### General DNA techniques

Standard recombinant DNA techniques were used for DNA preparation and analyses as described by Sambrook *et al*. [30]. Plasmid DNA preparations were isolated with QIAprep Miniprep Kit (Qiagen, Valencia, CA). PCR was carried out with Taq DNA polymerase (Invitrogen, Madison, WI). PCR products were purified with QIAquick PCR Purification Kit (Qiagen, Valencia, CA). Competence cells for *S. parasanguinis *electroporation were prepared as described previously [31].

### Mutagenesis

By using an *E. coli-S. parasanguinis *shuttle plasmid as template, inverse PCR was carried out to construct some in-frame deletion mutants of *gap3*. This plasmid contains *gap3 *and a downstream *gfp *tag (see Table 1). Site-directed mutagenesis was carried out by PCR utilizing the QuickChange XL Mutagenesis kit (Stratagene, La Jolla, CA). The mutagenic oligonucleotide primers are listed in Table 2. All mutations were sequenced at the DNA sequencing facility of the University of Vermont. Primary sequence data were analyzed using the BioEdit version 7.0.1.

**Table 2:** Oligonucleotide primers used in this study

Primer	Sequence	Reference or source
*gap3-gfp*-F	5'-CGGCCGTCGACATGACTAAACAGTTAATTTC-3'	This study
*gap3-gfp*-R	5'-CGCCGCGGTACCAATATATTCTATTAAATTTTTC-3'	This study
Δ62–67 Upstream	5'-GCAGACTCGAGAGAATGACGATCACCTTG-3'	This study
Δ62–67 Downstream	5'-CGGGCCTCGAGATACAAGGAAATAGATACCTTC-3'	This study
Δ144–157 Upstream	5'-GCCCGCCCTCGAGATCATAAATCATAAATTTTTG-3'	This study
Δ144–157 Downstream	5'-TATCTCTCGAGTATCTACGGGAACTGCTTCCG-3'	This study
Val33Arg-F	5'-CCAAGAGGATTTAAGGAATTTTTACAATCCACAGCTCGC-3'	This study
Val33Arg-R	5'-GCGAGCTGTGGATTGTAAAAATTCCTTAAATCCTCTTGG-3'	This study
Phe35His-F	5'-CCAAGAGGATTTAGTGAATCATTACAATCCACAGCTCGC-3'	This study
Phe35His-R	5'-GCGAGCTGTGGATTGTAATGATTCACTAAATCCTCTTGG-3'	This study
Asn37Ile-F	5'-GGATTTAGTGAATTTTTACATTCCACAGCTCGCATCAGGG-3'	This study
Asn37Ile-R	5'-CCCTGATGCGAGCTGTGGAATGTAAAAATTCACTAAATCC-3'	This study
Pro38Arg-F	5'-GTGAATTTTTACAATCGACAGCTCGCATCAGG-3'	This study
Pro38Arg-R	5'-CCTGATGCGAGCTGTCGATTGTAAAAATTCAC-3'	This study
Ser42Leu-F	5'-CAATCCACAGCTCGCATTAGGGAAGCCTATTG-3'	This study
Ser42Leu-R	5'-CAATAGGCTTCCCTAATGCGAGCTGTGGATTG-3'	This study
Asn54 Ile-F	5'-GTCACGAACAATTTATCAAGGTGATCGTCATTCTCC-3'	This study
Asn54 Ile-R	5'-GGAGAATGACGATCACCTTGATAAATTGTTCGTGAC-3'	This study
Pro62Gly-F	5'-GGTGATCGTCATTCTGGAGATCTTCCAATTC-3'	This study
Pro62Gly-R	5'-GAATTGGAAGATCTCCAGAATGACGATCACC-3'	This study
Asp63Val-F	5'-GGTGAT CGTCATTCTCCAGTTCTTCCAATTCTTATACAAGG-3'	This study
Asp63Val-R	5'-CCTTGTATAAGAATTGGAAGAACTGGAGAATGACG ATCACC-3'	This study
Leu64Arg-F	5'-CGTCATTCTCCAGATCGTCCAATTCTTATACAAGG-3'	This study
Leu64Arg-R	5'-CCTTGTATAAGAATTGGACGATCTGGAGAATGACG-3'	This study
Pro65Arg-F	5'-CGTCATTCTCCAGATCTTCGAATTCTTATACAAGG-3'	This study
Pro65Arg-R	5'-CCTTGTATAAGAATTCGAAGATCTGGAGAATGACG-3'	This study
Ile66Asn-F	5'-CGTCATTCTCCAGATCTTCCAAATCTTATACAAGG AAATAG-3'	This study
Ile66Asn-R	5'-CTATTT CCTTGTATAAGATTTGGAAGATCTGGAGAATGACG-3'	This study
Leu67Thr-F	5'-CGTCATTCTCCAGATCTTCCAATTACGATACAAGGAAATAG-3'	This study
Leu67Thr-R	5'-CTATTTCCTTGTATCGTAATTGGAAGATCTGGAGAATGACG-3'	This study
Leu75Ser-F	5'-GGAAATAGATACCTTTCAGAGTATCAGATTGATGCTGTACC-3'	This study
Leu75Ser-R	5'-GGTACAGCATCAATCTGATACTCTGAAAGGTATCTATTTCC-3'	This study
Arg59Leu-F	5'-CGAACAAATTATCAAGGTGATCTTCATTCTCCAGATCTTCC-3'	This study
Arg59Leu-R	5'-GGAAGATCTGGAGAATGAAGATCACCTTGATAATTTGTTCG-3'	This study

### Immunoblot analysis

Whole cell protein samples were determined by the Bicinchoninic Acid (BCA) Protein Assay. The quantified protein was boiled in sample buffer (0.0625 M Tris, pH 6.8, 2% SDS, 10% glycerol, 0.01% bromophenol blue) for 10 min before loading on acrylamide gels. Gels were run at 100 V for certain time period (depending on the MW of the proteins, for example, 2~2.5 hours for MW < 50 kDa; 6~8 hours for MW between 200 to 470 kDa). Separated proteins were transferred from gels onto nitrocellulose. Non-specific bands on nitrocellulose blots were blocked with a solution of TBST buffer (10 mM Tris-HCl, pH 7.5, 150 mM NaCl, 0.1% Tween-20) plus 5% non-fat dried milk (blocking buffer) for 1 h. Blots were then probed with various primary antibodies diluted in blocking buffer. Horseradish peroxidase-conjugated secondary antibody and chemiluminescent substrate (SuperSignal Pico Western Blot detection Reagents, Pierce, Rochford, IL) were used to detect bands that reacted specifically with primary antibodies.

### BactELISA

Cell surface Fap1 *of S. parasanguinis *mutants was determined using BactELISA (whole-bacterial-cell enzyme-linked immunosorbent assay [32]). Bacteria were immobilized and dried on 96-well microtitre plates by incubation at 37°C overnight. The wells were washed twice with TBST. Bovine serum albumin (BSA, 1% in TBS) was added to each well. The unbound BSA was removed by washes with TBST and the 1% BSA/TBS diluted MAbs were incubated with the bacteria at room temperature for 1 h. The wells were washed with TBST and incubated at room temperature for 1 h with 100 μl of 1:5,000 dilution of peroxidase-conjugate goat anti-mouse immunoglobulin (Sigma, St. Louis, MO) in 1% BSA/TBS. The wells were washed with TBST, and the enzymatic activity was determined by incubation with hydrogen peroxide in the presence of o-phenylenediamine in citrate buffer, pH 5.0. The reaction was stopped by the addition of 4 M sulphuric acid, and the color development was quantified by measurement of the absorbance at 490 nm using an Elx800 microtitre plate reader (Bio-Tek, Winooski, VT).

### Electron microscopy

Early exponential phase *S. parasanguinis *cell cultures (OD_470 _= 0.4) 5 ml were harvested by centrifugation. Cell pellets were washed twice with ice-cold PBS and resuspended in 100 μl PBS. A small aliquot (5 μl) of the bacterial suspension was diluted in PBS was applied to 400 mesh copper grids coated with a thin carbon film. The grids were washed by PBS buffer. The samples were negatively stained as described by Ruiz *et al *[33] with 2% phosphotungstic acid, pH 7 with NaOH or with NanoW (Nanoprose, Plano, TX). The last drop was left on the grids for 30 sec. Finally, the excess liquid was wicked off and the grids were fast air-dried. The grids were observed on a Tecnai12 Philips electron microscope (FEI, The Netherlands) equipped with a LaB6 cathode (Kimball, Wilton, NH) operated in point mode [34], and a 14 μm 2048 × 2048 CCD camera (TVIPS, Germany). Images were recorded at an accelerating voltage of 100 kV and nominal magnifications in the range of 40000–70000X under low dose conditions on either film (S0–163 Kodak) or the CCD camera. Images were converted to SPIDER format [35] and high-pass filter to remove the background variations.

### SHA binding experiment

An *in vitro *tooth model, Saliva-coated hydroxylapatite (SHA) was used to test the effects of *gap3 *mutagenesis to *S. parasanguinis *adhesion ability. This method was described previously [24]. Briefly, [^3^H]-thymidine-labeled bacteria (5 × 10^8^) in adhesion buffer (67 mM phosphate buffer, pH6.0) were sonicated for 15 s at 85 W using an ultrasonic cuphorn system (Heat systems-Ultrasonics, Farmingdale, NY). 1 ml of sonicated bacteria (in triplicate) were added to 10 ml scintillation vials containing SHA and incubated for 1 h at 37°C with gentle shaking. The beads were allowed to settle and the supernatant fluids were removed. The beads were washed 3 times with adhesion buffer. The amounts of unbound bacteria in the supernatant fluids and bacteria bound to SHA were determined in a Wallac 1400 liquid scintillation counter.

## Authors' contributions

ZP, PFT and HW developed methods. ZP, HW and QC designed the experiments. ZP, MZ and BS performed the experiments with bacteria. TR carried out the electron microscopy experiments and interpreted the results. ZP analyzed data from assays and drafted the manuscript. PFT, TR and HW revised the manuscript. All authors read and approved the final manuscript.
